# Are Second Person Masculine Generics Easier to Process for Men than for Women? Evidence from Polish

**DOI:** 10.1007/s10936-022-09859-7

**Published:** 2022-03-18

**Authors:** Agnieszka Szuba, Theresa Redl, Helen de Hoop

**Affiliations:** 1 Centre for Language Studies, Radboud University, Postbus 9103, 6500 HD Nijmegen, the Netherlands; 2 Max Planck Institute for Psycholinguistics, Nijmegen, the Netherlands

**Keywords:** Masculine generics, Second person, Addressing, Gender marking mismatch

## Abstract

In Polish, it is obligatory to mark feminine or masculine grammatical gender on second-person singular past tense verbs (e.g., *Dostałaś list* ‘You received-F a letter’). When the addressee’s gender is unknown or unspecified, masculine but never feminine gender marking may be used. The present self-paced reading experiment aims to determine whether this practice creates a processing disadvantage for female addressees in such contexts. We further investigated how men process being addressed with feminine-marked verbs, which constitutes a pragmatic violation. To this end, we presented Polish native speakers with short narratives. Each narrative contained either a second-person singular past tense verb with masculine or feminine gender marking, or a gerund verb with no gender marking as a baseline. We hypothesised that both men and women would read the verbs with gender marking mismatching their own gender more slowly than the gender-unmarked gerund verbs. The results revealed that the gender-mismatching verbs were read equally fast as the gerund verbs, and that the verbs with gender marking *matching* participant gender were read faster. While the relatively high reading time of the gender-unmarked baseline was unexpected, the pattern of results nevertheless shows that verbs with masculine marking were more difficult to process for women compared to men, and vice versa. In conclusion, even though masculine gender marking in the second person is commonly used with a gender-unspecific intention, it created similar processing difficulties for women as the ones that men experienced when addressed through feminine gender marking. This study is the first one, as far as we are aware, to provide evidence for the male bias of second-person masculine generics during language processing.

## Introduction

Imagine that you lost your cat. In Standard English, you can show the picture of the cat and ask *Have you seen this cat?,* in this exact same form, to anyone. You can ask any one of your neighbours, and you can also write it on posters and hang them around the neighbourhood in order to address all passersby. This would work slightly differently in Polish, where gender marking is obligatory on past tense verbs. In the first person, gender marking needs to match the gender of the speaker, and in the second person it needs to match the gender of the addressee. Furthermore, although Polish has a neuter gender, only masculine and feminine gender marking are allowed in the first and second person.^1^ Thus, (1a) should be used to address your female neighbours, while (1b) should be used to address your male neighbours.^2^

**Table:** 

(1)	a	*Widział-a-ś*	*tego*	*kota?*
		saw-f-2sg	this	cat
		‘Have you [female] seen this cat?’		
	b	*Widział-e-ś*	*tego*	*kota?*
		saw-m-2sg	this	cat
		‘Have you [male] seen this cat?’		

Which form should be put on a poster that aims to address passersby of any gender, though? In such situations—when the addressee can be of any gender—Polish speakers employ two main strategies. The first one is using the masculine form of the verb, which in these situations is commonly understood to be gender-unspecific. Alternatively, it is possible to use *both* the feminine and the masculine verb form (e.g., *Widziałeś/aś tego kota?)*. The feminine form of the verb on its own, however, cannot be used with a gender-unspecific meaning.

The gender-unspecific use of the masculine but not the feminine gender marking is common across languages and constitutes what is often termed *masculine generic language* (e.g. De Backer & De Cuypere, 2012; Gastil, 1990; Irmen & Rossberg, 2004; Stahlberg et al., 2001). The aim of the present study is to investigate whether Polish-speaking men and women process being addressed with second person masculine generic verbs equally well, or whether these masculine generics result in a processing disadvantage for women. This will be tested through a self-paced reading experiment.

## Research on the Interpretation of Masculine Generics

Masculine generics are common across languages and linguistic structures (e.g., Hellinger & Bussmann, 2001). For example, the pronoun *his* in (2) is intended to refer to a person of any gender. Similarly, in languages that mark grammatical gender on nouns, many nouns describing people (so-called role nouns) exist both in a grammatically feminine and a masculine version, but the masculine form can also be used more broadly, such as to describe a semantically generic person in the singular or to a mixed-gender group in the plural.

(2) Every student should hand in his master’s thesis on time.

In recent decades, there have been growing concerns about the possibility of masculine generics being at odds with the pursuit of gender equality, for example by reducing women’s visibility or perpetuating the notion of “male as norm” (e.g., Stahlberg, Braun, Irmen, & Sczesny, 2011). Multiple experimental studies have provided scientific evidence that supports these concerns (e.g., Bojarska, 2011; Garnham & Yakovlev, 2015; Gastil, 1990; Gygax et al., 2008; Irmen, 2007). Specifically, studies on masculine generic role nouns and third person pronouns show that descriptions of people that contain masculine generics are more likely to evoke male mental imagery compared to female imagery (but see also Cole et al., 1983 and Redl et al., 2018 for null results).

A large subset of those studies approached this question from an online processing perspective using methods like self-paced reading, eye-tracking, sentence evaluations or event-related potentials (ERPs) (e.g., Garnham & Yakovlev, 2015; Gygax et al, 2008; Irmen, 2007; Misersky, Majid, & Snijders, 2019). For example, Gygax et al. (2008) used stimuli such as (3) in English, German, and French. In the German and French versions, the noun phrase (e.g., *social workers*) was presented in its masculine form (e.g., *Sozialarbeiter*). After reading each text, the participants had to indicate whether the second sentence was a sensible continuation of the first one. The results revealed that participants took longer to accept female continuations (i.e., *some of the women…*) than male continuations in German and French, but both continuations were accepted equally fast in English, which does not mark gender on nouns.

(3) The social workers were walking through the station. Since sunny weather was forecast several of the [women/men] weren’t wearing a coat.

Such findings show that although masculine generic nouns and pronouns are intended to be gender-unspecific, they are not necessarily interpreted as such. Furthermore, some studies provide evidence of negative consequences that this male bias of masculine generics may have. For example, in a German hiring simulation study, Horvath and Sczesny (2016) found that female applicants for high-status positions were perceived as a worse fit for the job when the job title was in its masculine noun form, compared to a masculine-feminine pair. A study by Vervecken et al. (2013) with German- and Dutch-speaking children found that when a stereotypically masculine profession was introduced using only the masculine form, women were perceived as less successful in that profession. Moreover, girls expressed less of a desire to perform said professions in the future. These findings, together with the results of the online processing studies, provide substantial evidence that third person masculine generic nouns and pronouns favour men over women. However, little is known about the effects of other masculine generics.

## Second Person Masculine Generics

While the existing research on masculine generics has largely focused on the third person, some languages also use masculine generics in the second person. Polish, like other Slavic languages, marks gender on verbs in the past tense (Migdalski, 2018). This obligatory gender marking results in masculine generics in the second person singular, such as in the example shown at the beginning of this article. These second person masculine generics, just as the ones in the third person, raise the question of whether they are interpreted in the way that they are intended—as gender-unspecific—or whether they are associated with men nonetheless. If the latter is found to be the case, this may give rise to similar concerns as those about third person masculine generics. For example, while third person masculine generics may reduce the visibility of women by making the reader or hearer think of the described referent as male, second person masculine generics may make women feel less addressed than men by statements formulated using second person masculine generics.

As far as we know, the topic of second person masculine generics was only addressed in one study (Kricheli-Katz & Regev, 2021) on Hebrew—a language that marks gender on verbs and where the masculine gender can also be used generically. They found that both men and women performed worse on a math test when the questions addressed them using gender marking that mismatched their own gender. Similar results were found for reading comprehension and word association tests, though for those tests the effect was not statistically significant for the male participants. Gender marking was also associated with time spent on the math test—more time was spent when the gender marking *matched* the test taker’s gender. More time spent on the tests, in turn, predicted higher scores. Additionally, the participants were asked to rate their agreement with the statement “science is for men” after taking the math test. Both men and women agreed more with the statement when they were addressed using masculine marking in the test. These results suggest that masculine marking activates associations with men, which may cause women to see themselves as a less prototypical test taker and to reduce their feelings of self-efficacy. This, in turn, may affect their motivation, which would be reflected through shorter time spent on the tests and through lower scores. However, the exact process that underlies these effects is not completely clear. What remains unknown is whether these gendered associations are activated during language processing and whether they affect processing speed.

The aim of the present self-paced reading experiment was to test the hypothesis that masculine generic second person verbs activate associations with men. We compared men and women’s processing of second person singular masculine generic verbs, as well as their processing of verb forms without gender marking. If being addressed using verbs with masculine marking is more difficult to process for female addressees, this would indicate that second person masculine generics are not interpreted as gender-unspecific, but as forms associated with male addressees.

In addition, we included stimuli containing second person verbs with feminine marking. We expected that being addressed using feminine marking would cause significant processing difficulties for men, as feminine marking is never used when addressing men. As such, we were able to compare the effect on men of being addressed using feminine marking with that of women being addressed using masculine marking. Furthermore, including the condition of men being addressed using feminine marking served to verify whether this type of violation would slow down reading in the first place. As will be discussed in the following section, there is not much existing research into the processing of mismatches between a linguistic form and the addressee (or speaker) identity. Because of that, we could not simply assume that men would slow down in reading when addressed using feminine marking.

## The Processing of Inappropriate or Anomalous Forms in the Second and First Person

The main hypothesis of this study—that women would experience a processing cost when addressed using verbs with masculine marking—rests on the assumption that people’s language processing would be sensitive to being addressed with linguistic forms that are in some way not in line with their identity. However, while this idea may be intuitively true, empirical research in support of it is scarce.

Many studies in the *third* person have shown that ungrammatical or infelicitous gender marking does negatively affect reading (e.g., Dank et al., 2015; Irmen & Schumann, 2011; Keating, 2009). However, in the third-person studies the gender marking mismatch always existed on the level of the text. By contrast, the referent of second (and first) person is always deictic (Levinson, 2006), meaning that a mismatch only occurs when we consider the addressee (or speaker) identity. This also means that first or second person gender marking can only be evaluated in terms of pragmatic appropriateness, rather than grammaticality. Previous syntactic analyses of gender marking predict that free, referential uses of gender marking will be evaluated for appropriateness within the broader sociopragmatic context (e.g. Conrod, 2019; Sigurðsson, 2018). However, it is less clear how the evaluation process would work, and whether and how it would be reflected during reading. It is therefore of interest to investigate cases when the gender marking is considered inappropriate according to the pragmatic conventions in Polish (men being addressed using feminine gender marking), as well as those that can be considered pragmatically appropriate, but which require a more extensive consideration of the context (women being addressed using masculine marking).

While we are not aware of any reading studies investigating this topic, a number of ERP studies suggest that people *are* sensitive to mismatches between speaker or addressee identity and the linguistic form used. In a study on Slovak, Hanulíková and Carreiras (2015) played recordings of first- and third-person utterances spoken by a man or a woman. The utterances contained a past-tense verb, which, like in Polish, needs to be marked for gender. They found that when the gender marking on the first-person verb mismatched the speaker’s inferred gender (e.g., a feminine voice saying *Susedia sa nahnevali, lebo som kradol slivky* ‘The neighbours were upset because I stole-M plums’), an N400 effect was elicited. By contrast, a gender mismatch in the third person resulted in a P600 effect. This shows that listeners were sensitive to both types of violations, even though they were processed in different ways.

The authors interpreted this finding as indicative of listeners creating predictions regarding grammatical forms based on speaker characteristics. Other studies also suggest that such predictions are made not only with regard to grammatical form, but also based on the semantic content of the utterances. For example, Lattner and Friederici (2003), as well as Van Berkum, Van den Brink, Tesink, Kos, and Hagoort (2008) found that when speakers produced semantic content that went against what was stereotypically associated with their age, gender, or socioeconomic status—such as a woman saying ‘I like to play soccer’—it elicited a P600 (Lattner & Friederici, 2003) or N400 (Van Berkum et al., 2008) effect in the listeners.

Additional evidence comes from a study on Mandarin Chinese honorifics by Jiang et al. (2013). They found that when participants followed an exchange between two interlocutors, they were sensitive both to a disrespectful use of the Chinese honorific (e.g., a student addressing a professor with the informal pronoun *ni/ni-de*), as well as an over-respectful honorific use (e.g., a professor addressing a student with the formal pronoun *nin/nin-de*). Interestingly, the disrespectful use constitutes a pragmatic violation in Chinese and can only be interpreted as an insult or a mistake when it occurs. The over-respectful use, on the other hand, is considered atypical, but not always inappropriate. These two types of mismatches were found to elicit different ERP effects in the listeners. Only the disrespectful honorifics elicited an early effect (the N400). On the other hand, both types of mismatches elicited late effects—either a late positivity effect when the pronoun was over-respectful or a late negativity effect when the pronoun was disrespectful. The authors relate the late positivity effect to that of non-literal language processing (re-interpreting the over-respectful pronoun as constituting a joke or irony), and the late negativity effect to the mental revision or replacement of the disrespectful pronoun with the respectful one, after coming to the conclusion that its use was most likely a mistake.

It should be noted that while there are undoubtedly differences between honorifics and gender marking, multiple parallels have been drawn between them. Conrod (2019) used syntactic, sociolinguistic, and pragmatic data to analyse English third person singular pronouns. They argued that gender marking that reflects social, rather than grammatical, gender behaves similarly to honorifics in several ways. Both are encoded in functional, rather than lexical, categories and are subject to syntactic processes such as Agree. At the same time, when used referentially, both can only be evaluated based on appropriateness, by taking into account the sociopragmatic context, rather than based on truth conditions. Both can also be dynamically constructed, such that different gender or honorific marking can be used to refer to the same person depending on the context. While these characteristics have more commonly been acknowledged in the study of honorifics rather than gender, Conrod showed that analysing variation in English third-person pronouns with these characteristics in mind explains much of the sociopragmatic variation in natural language use. Therefore, the findings from the study on honorifics are perhaps more relevant to the current study than would have previously been assumed.

Together, the ERP studies show that people are sensitive to not only pragmatic violations in the first and second person, but also to language that is merely unexpected or atypical, such as stereotype-incongruent semantic content or over-respectful honorific use. Women being addressed using masculine marking in Polish may also fall under the latter category, since the default way to address women, and the only acceptable way in many situations, is by using feminine marking. However, while the previous findings back up our predictions to some extent, there are several factors that prevent us from assuming that the same result pattern will be found in the present study. None of the previous studies were concerned with gender marking in the second person, and in none of them was the *participant* the target of the pragmatic violation.

Perhaps the largest difference between the previous studies and the current one is in the methodology. ERP findings have often been replicated with reading-time methods and vice versa (Mitchell, 2004), for example in studies on the processing of non-literal language (e.g., Brisard et al., 2001; Filik & Moxey, 2010; Coulson & van Petten, 2007; Regel et al., 2010) or in studies on the role of discourse context in the processing of syntactic ambiguities (e.g., Altmann & Steedman, 1988; Altmann et al., 1992; van Berkum et al., 1999). However, it is not always the case that a process that requires more neural processing resources (as indicated by ERPs) also requires more time (Coulson, 2007). This does not necessarily mean that ERPs are a more sensitive method—as van Berkum (2004) argues, there are methodological challenges specific to the ERP method which can cause an existing effect to not be detected. It does, however, reflect the fact that due to the specificities and intricacies of different online language processing methods, we cannot assume that an effect found using one method will also appear when using another method. This is also a reason why we believe that our choice of a self-paced reading method offers a valuable contribution to the body of research comprising the studies mentioned above, as applying different methods to related research questions can help to clarify the nature and ubiquity of certain effects. Furthermore, compared to ERPs, self-paced reading is a simpler and cheaper method. If we find that effects similar to the one hypothesised in this study are sensitive to the self-paced reading method, it may allow more researchers, who may not have the resources to conduct ERP studies, to contribute to this field. Of course, we are also aware of the advantages that ERPs offer, especially with regards to providing insights into the nature and timing of an effect (van Berkum, 2004). However, for research questions that are primarily concerned with *whether* an effect is present (such as in the current study), self-paced reading could be an attractive option.

## Current study and Hypothesis

The aim of the current study can be summarised by the following research question: Is Polish speakers’ reading speed negatively affected when they are addressed using a second-person singular verb with gender marking that mismatches their own gender? This question can be divided into two sub-questions: (1) Is women’s reading time negatively affected when they are addressed using a second-person singular verb with masculine gender marking, even in contexts where the use of masculine marking is considered appropriate?; (2) Is men’s reading time negatively affected when they are addressed using a second-person singular verb with feminine marking?

A self-paced reading experiment was conducted in order to answer this question. Participants were shown three-sentence stories, such as the one in (4).

(4) Wyobraź sobie, że wyprowadzasz się do Norwegii na pół roku. Gdy już sprawdziłeś dojazd na lotnisko, zamierzasz pożegnać się z rodziną. Jednak w tym momencie babcia zaczyna pakować ci zapasy jedzenia do walizki.

‘Imagine that you are moving to Norway for half a year. Once you checked the transport to the airport, you want to say goodbye to your family. But in that moment, your grandmother starts to pack food into your suitcase.’

The first verb of the second sentence (e.g., *sprawdziłeś* ‘checked’) was manipulated between a past tense verb with masculine marking, a past tense verb with feminine marking, and a gerund verb which requires no gender marking. We hypothesised that both men and women would read the verbs with gender marking mismatching their own gender more slowly than the gerund verbs with no gender marking. However, we expected that the increase in reading time would be larger for men, as using feminine marking to address men would be very unexpected, since it constitutes a pragmatic violation. By contrast, the use of masculine marking in the context of the experiment does not constitute a pragmatic violation, as it is only a violation if the only possible addressees of the text are female. The participants, however, had no reason to believe that this was the case.

## Method

### Participants

The study was approved by the Radboud University’s Ethics Assessment Committee Humanities of the Faculty of Arts and the Faculty of Philosophy, Theology and Religious Studies. In total, 88 participants (34 men) were tested in the self-paced reading experiment. They were recruited through various means, such as personal contacts, university mailing lists, or posters. The participation requirements were that the participant was a native Polish speaker, did not have dyslexia or other reading problems, and was between 18 and 40 years old. Additionally, two exclusion criteria were applied: answering less than 75% of the comprehension questions correctly and/or guessing the purpose of the experiment.

### Materials

The experiment consisted of 48 stimuli, each in three versions. A full set of glossed stimuli (in the version with masculine gender marking) can be found in “Appendix A”. Additionally, Table 1 shows the crucial part of the example stimulus from (4) in each version. Each stimulus consisted of three sentences. The first sentence always started with the gender-neutral phrase *Wyobraź sobie, że…* ‘Imagine that…’ and a second person singular verb. It introduced a certain event that the participant was asked to imagine themselves to be taking part in. In the experimental versions (with gender marking), the second sentence opened with *Gdy już* ‘Once’ followed by a second person singular past tense verb with masculine or feminine marking. In the control version, a gerund verb was used instead of a past tense verb, and *Gdy już* ‘Once’ was replaced with *Po* ‘After’. The critical verb was followed by a noun, which in the experimental versions had accusative case marking, while in the control versions it had genitive case marking. The third sentence always opened with *Jednak w tym momencie…* ‘But in that moment…’ and introduced an unexpected event which served to round off the story. The verb and the noun following it were always the same length in the masculine and the feminine form, and most of the time also in the gerund form. When the length differed in the gerund form it was by one or two characters, and most of the time the gerund form was the longer one.

**Table 1:** The beginning of the second sentence of an example stimulus in versions with feminine, masculine, and no gender marking

Feminine	*Gdy*	*już*	*sprawdził-a-ś*	*dojazd*	*na*	*lotnisko*
	When	already	checked-f-2sg	transport. acc	to	airport
Masculine	*Gdy*	*już*	*sprawdził-e-ś*	*dojazd*	*na*	*lotnisko*
	When	already	checked- m-2sg	transport. acc	to	airport
Control	*Po*	*sprawdzeniu*	*dojazdu*	*na*	*lotnisko*	
	After	checking	transport. gen	to	airport	

The stimuli were pre-tested in order to ensure that the described scenarios do not evoke gender stereotypes. Gender stereotypes could result in different reading patterns depending on the participant’s gender and therefore constitute a confounding factor. We initially created 62 stimuli out of which 48 stimuli with the most gender-neutral ratings were selected based on the pre-test that was conducted through a Qualtrics (www.qualtrics.com/) survey. The control versions of the stimuli were re-written in the first person, and the respondents were told that each of the stories was told by a different person. Their task was to indicate on a 7-point scale how likely they thought it was that each story was told by a man or a woman. Scale direction was counterbalanced and the male and female respondents were equally distributed between the two versions. The data of 24 women and 19 men between the ages of 18 and 46 (*M* = 28.19) contributed to the analysis. All data were coded so that 1 corresponded to the most feminine rating, and 7 to the most masculine rating. The mean rating of the 62 items was 3.91 (*SD* = 1.19), ranging from 2.88 to 5.01. Based on the pre-test results, we first discarded items with the highest standard deviation (> 1.3) and then selected the 48 items closest to 4, indicating a neutral rating. The final sample had a mean rating of 4 and a standard deviation of 0.34, with mean ratings ranging from 3.42 to 4.88.

In addition to the 48 stimuli, 48 fillers were created. Eighteen of those fillers were also second-person narratives asking the participant to imagine themselves as a protagonist in a certain scenario. However, unlike in the stimuli, the unexpected action in the fillers was described in the *second* instead of the third sentence. In the stimuli, the second sentence contained the crucial verb. However, from a reader’s point of view, this sentence was not the most prominent one, as it mainly set up the stage for the unexpected action of the third sentence. This introduced the risk that the participants might learn that the second sentence was not that crucial to the story and might read it less carefully. For this reason, in the eighteen fillers, the second sentence was the one that contained an unexpected event. The remaining 36 fillers also asked the participant to imagine something, but only from the point of view of an observer rather than an actor. The role of these fillers was to allow the participants to take a break from imagining themselves doing something.

**Fig. 1:**
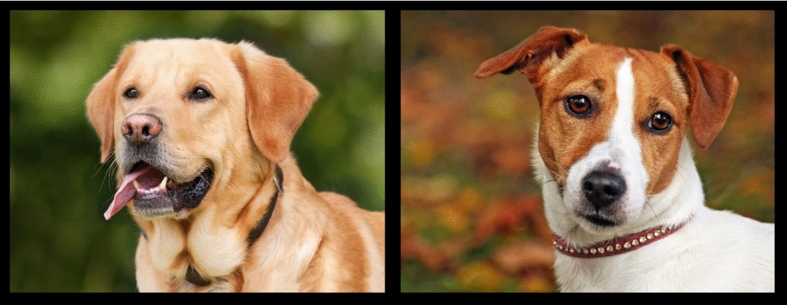
An example of a pair of images shown after a stimulus in the self-paced reading experiment. The participants’ task was to pick the image which more closely resembled what they had imagined while reading a particular story

One quarter of stimuli was followed by a comprehension task in order to ensure careful reading. Participants had to indicate whether a presented statement about a stimulus they had just read was correct or incorrect. Additionally, after another quarter of the stimuli, participants had to complete an image task. A pair of images related to the content of the previous text were shown on the screen. An example of such an image pair can be seen in Fig. 1. The task of the participant was to choose the image that reflected more accurately what they imagined while reading that text. The main goal of the image task was to mimic real-world contexts of being addressed, where there is usually a purpose to addressing someone. The task was also thought to increase the chances that the participant did what the stimuli texts asked them to do—imagine themselves as the protagonist or, in other words, self-ascribe the second person reference. Furthermore, we intended the image task to divert the participants’ attention from the purpose of the experiment.

### Design

As can be seen in Table 2, all of the experimental items with masculine gender marking were shown before all of the ones with feminine marking. The reason for this is that if women are addressed using masculine marking *after* having been addressed using feminine marking, the use of the masculine marking may not be interpreted as gender-unspecific anymore. This is in line with a study by Gygax and Gabriel (2008) that found that more male bias was associated with masculine role nouns in French (e.g. *musiciens* ‘musicians-M’) when the participants were previously presented with feminine nouns (e.g. *musiciennes* ‘musicians-F’). As the aim of this study is to investigate whether women experience a processing cost *even when the use of masculine gender marking is used in a gender-unspecific way*, it is important that the masculine marking is interpreted as such. By contrast, feminine gender marking can never be interpreted as gender-unspecific, so men should interpret it as inappropriate regardless of whether it is preceded by items with masculine marking or not. Four lists of stimuli were created. Each list had a different set of 12 stimuli in the versions with and without gender marking in each block. An equal number of male and female participants was assigned to each list. The order of items within blocks was pseudorandomized.

**Table 2:** The design of the self-paced reading experiment

Block 1		Block 2	
Number	Type of item	Number	Type of item
12	Masculine gender marking on verb	12	Feminine gender marking on verb
12	No gender marking on verb	12	No gender marking on verb
24	Fillers	24	Fillers

### Procedure

The experiment was conducted at the Institute of English Studies of the University of Wrocław in Poland. A Dell laptop with a screen resolution of 1920 × 1080 was used. For both the stimuli and the fillers, the first sentence was presented all at once, and the rest of the text was presented one word at a time. A centred, non-cumulative display was used. Stimuli were presented in a Deja Vu Sans Mono size 32 font, in white on a black background. Trials were separated by a fixation cross which stayed on the screen for 300 ms before the participant could proceed. A button box was used to collect responses.

Participants were tested individually. Before beginning the experiment, they gave signed consent, were given written as well as verbal instructions in Polish, and shown an example trial. Next, they were presented with three practice items to go through on their own. There were three breaks during the experiment. A longer break took place in between the first and the second block. Additionally, two shorter breaks were scheduled halfway through each block. After the experiment was over, the participants completed an exit questionnaire and were given a 25 złotych (approximately 6 euros at the time of testing) coupon to a chain of bookstores. The exit questionnaire collected demographic data, as well as asked the participant to guess the purpose of the experiment. On average, the experiment took 50 minutes.

## Results

The data of six participants were excluded from the analysis. One participant was excluded because they were not a native Polish speaker. Another one was given a faulty version of the stimulus file. In addition, after checking the answers to the comprehension questions, four additional participants were excluded because they answered less than 75% of the questions correctly. The remaining sample consisted of 82 native Polish speakers (32 men) between the ages of 18 and 31 (*M* = 22.56, *SD* = 2.76). The majority (68) of the participants were students. Most of those who were not students (10) had completed higher education. None of the participants guessed the purpose of the experiment. Before performing the analysis, all data points reflecting reading times on the verb or noun shorter than 150 ms or longer than 3000 ms were removed. Responses quicker than 200 ms in self-paced reading experiments are most likely caused by unintentional button presses (Jegerski, 2014). An upper cutoff of 3000 ms has been previously applied in self-paced reading studies (Havik, Roberts, van Hout, Schreuder, Haverkort, 2009; Roberts & Felser, 2011). The removal of extreme data points resulted in the loss of 0.003% data points for both the verb and the noun. The remaining data were log-transformed in order to achieve a normal distribution of the data. The reading times of the verb and the noun were modelled using a linear mixed model constructed in R (R Core Team, 2018) using the lme4 package (Bates, Maechler, Bolker & Walker, 2015). Block, gender marking, participant gender, as well as their interactions were entered as fixed effects. The levels of the factors were coded using sum contrasts. Table 3 shows the codes that were used.

**Table 3:** Contrast coding for the fixed factors

Factor	Contrast coding	
	− 1	+ 1
Block	1	2
Gender marking	Unmarked	Marked
Participant gender	Male	Female

Participant and item were entered as random effects. The full random structure permitted by the design was initially used, but this resulted in the model failing to converge. The model was simplified by suppressing the correlation parameters, and then gradually removing the smallest variance components until the model successfully converged. As a result, the random slopes that were used in the final models were block as a random slope for participant, and, only in the noun model, the interaction between gender marking and participant gender as a random slope for item. P-values were calculated for each of the coefficients using the lmerTest package (Kuznetsova, Brockhoff, & Christensen, 2017). The descriptive statistics (means and standard deviations) for the reading time on the verb and the noun can be found in “Appendix B”. The full output of the linear mixed models can be found in “Appendix C”.

### Main Region of Interest: Verb

**Fig. 2:**
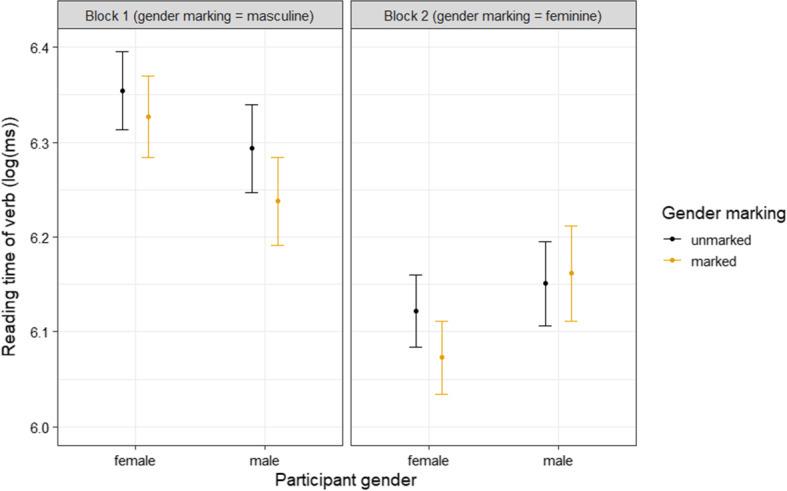
Mean reading time of verbs with and without gender marking across conditions. Error bars represent 95% confidence intervals

The output of the linear mixed model revealed an unexpected main effect of block: participants read faster in the second block compared to the first (β = − 0.09, *SE* = 0.01, *t* = − 7.43, *P* < 0.0001). There was also a main effect of gender marking: verbs with gender marking (second person past tense verbs) were read faster than the gerund verbs without gender marking (β = − 0.02, *SE* = 0.01, *t* = − 3.15, *P* = 0.002). Additionally, there was a two-way interaction between block and participant gender (β = − 0.03, *SE* = 0.01, *t* = − 2.87, *P* = 0.005): while both men and women read faster in the second block, this difference was larger for the female participants. In other words, the increase in reading speed between block 1 and block 2 was larger for the female participants, who went from being addressed with the gender marking that *mismatches* their gender in block 1, to the gender marking that *matches* their gender in block 2. For the male participants, for whom the change was in the opposite direction (matching gender marking to mismatching gender marking), the increase in reading speed between block 1 and block 2 was not as large. Finally, there was a three-way interaction between gender marking, block and participant gender (β = − 0.01, *SE* = 0.01, *t* = − 2.29, *P* = 0.02). This interaction showed that the effect of gender marking was different for men and women across blocks. Figure 2 shows the mean reading times across conditions, which helps us to understand this interaction effect. We can see that while there was an overall effect of verbs with gender marking being read faster than verbs without gender marking (the main effect), this effect was more pronounced when the gender marking matched the participant gender, so in block 1 for men, and in block 2 for women. While we predicted a three-way interaction to occur, it is of a different nature than was predicted by the hypothesis. We had hypothesised that verbs with gender marking *mismatching* the participant gender would be read *slower* than the control verbs with no gender marking. However, as we can see in Fig. 2, there was very little difference in the reading times between those verbs and the control verbs. Instead, there is a difference between the control verbs and the verbs with gender marking *matching* the participant gender—the latter were read quicker. What can also be seen from the bar plot in Fig. 2, is that the two-way interaction between block and participant gender still holds when we take into account gender marking—across both levels of gender marking, the decrease in reading time between block 1 and block 2 was larger for female, compared to male, participants.

### Spillover Region: Noun

**Fig. 3:**
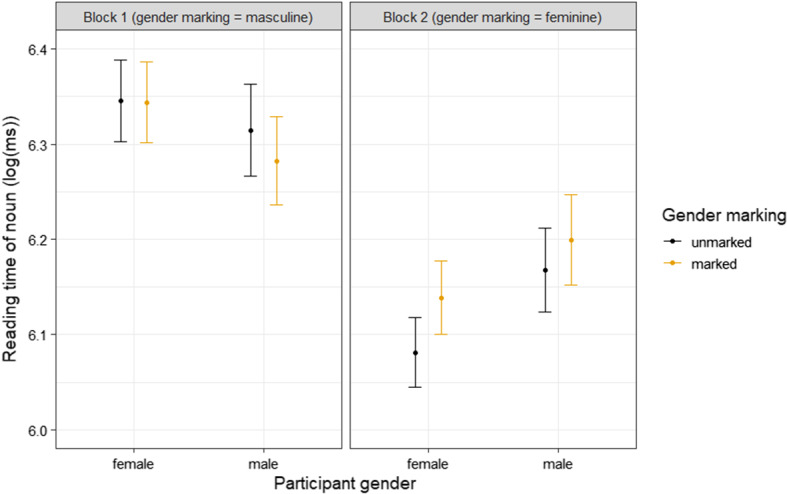
Mean reading time of noun following verbs with and without gender marking across conditions. Error bars represent 95% confidence intervals

The output of the linear mixed model for the noun also revealed a main effect of block: participants read faster in the second block (β = − 0.09, *SE* = 0.01, *t* = − 8.29, *P* < 0.0001). A two-way interaction between block and participant gender was found again as well: the difference in reading speed between the two blocks was more pronounced for women than for men (β = − 0.03, *SE* = 0.01, *t* = − 2.9, *P* = 0.005). Additionally, unlike on the verb, a two-way interaction between gender marking and block was found (β = 0.02, *SE* = 0.005, *t* = 3.14, *P* = 0.002). It shows that, on average, there was a tendency for the nouns following verbs with masculine marking (block 1) to be read faster, and the nouns following verbs with feminine marking (block 2) to be read slower, than the nouns following the control verbs. A three-way interaction between participant gender, block, and gender marking was not found for the noun. This is illustrated in Fig. 3.

To summarise the results, a main effect of block, and a two-way interaction between block and participant gender were found for both the verb and the noun. Both men and women read faster in block 2 than in block 1, but the increase in speed was larger for women, who went from being addressed using gender marking *mismatching* their gender to gender marking *matching* their gender. For the verb, there was also a main effect of gender marking: verbs with gender marking were read generally faster than those without gender marking. There was also a three-way interaction between participant gender, block, and gender marking. The advantage of verbs with gender marking compared to those without was larger when the gender marking matched participant gender (men in block 1, women in block 2). For the noun, these two latter effects were not found. Instead, a two-way interaction between gender marking and block was found: nouns following verbs with masculine marking were read faster than the control nouns, and nouns following verbs with feminine marking were read slower than the control nouns by both men and women.

## Discussion

The aim of the self-paced reading experiment was to find out whether Polish speakers experience a processing cost, reflected in slowed down reading, when addressed using verbs with gender marking that mismatches their own gender. For the female participants, the question was specifically whether they would experience a processing cost when the use of masculine gender marking constitutes a masculine generic and is thus considered felicitous. A three-way interaction between block, gender marking, and participant gender was predicted. When the past tense verbs had masculine gender marking (in block 1), women, but not men, were expected to take longer to read those verbs than the control gerund verbs. In block 2, where the past tense verbs had feminine gender marking, longer reading times compared to the control verbs were expected from men but not from women. A three-way interaction was indeed found on the verb, though it showed a slightly different pattern than the one hypothesised. The verbs with gender marking *mismatching* participant gender were *not* read more slowly than the control verbs. However, the verbs with gender marking *matching* participant gender were read faster than the control verbs. In other words, while a gender mismatch did not slow down reading compared to the control verbs, a gender match sped it up. Although the three-way interaction appeared differently than hypothesised, it still reveals a gender disparity. If masculine gender marking speeds up processing for men but not for women, it means that women are disadvantaged compared to men when masculine gender marking is used. Similarly, men are disadvantaged compared to women when feminine marking is used.

We should also keep in mind that we cannot necessarily talk about a processing advantage of gender-matching verbs and a processing disadvantage of gender-mismatching verbs as conceptually separate effects. Processing advantage and processing disadvantage are relative terms, and whether something shows up as one or the other in an experiment simply depends on what the comparison is. The fact that the three-way interaction manifested itself differently than hypothesised can most likely be explained by a baseline reading time difference between gerund verbs and second person singular past tense verbs, which we did not anticipate. We assumed that if there was no effect of gender marking, second person singular past tense verbs and control verbs would be read equally fast. However, a main effect of gender marking on the verb showed that the control verbs were generally read more slowly than the experimental verbs. While we did not consider the possibility of a baseline difference when forming the hypothesis, it does not come as a surprise, as the control and experimental verbs are quite different verb forms (second-person singular vs. gerund). The possibility of a baseline difference could not be avoided in this experiment, as verb forms identical to the experimental ones except without feminine or masculine gender marking do not exist in Polish.

There is one other aspect in which the pattern found by the three-way interaction differs from the hypothesised one. We expected that the processing disadvantage caused by incongruent gender marking would be larger for men than for women, since for men the incongruence constitutes a pragmatic violation. However, this was not the case. This suggests that whether the gender marking can function generically or not does not affect its (initial) processing. In other words, it seems that incongruent gender marking on the verb affects processing for women to the same extent as it does for men, despite the fact that the former does not constitute a pragmatic violation and the latter does.

Another effect that was found on the verb, as well as on the noun following it, is a two-way interaction between gender and block. It showed that while both men and women read faster in the second block (most likely due to practice effects), this difference was larger for the female participants. We know from the previously discussed three-way interaction that women read the (feminine) gender-marked verbs in block 2 faster than the (masculine) gender-marked in block 1. However, the two-way interaction shows that women also read the control verbs faster in block 2 than in block 1. It is unclear how this effect could be explained. Perhaps, for whatever reason, women are more susceptible to practice effects than men. It is also possible that the feminine gender marking had a more global positive effect on women’s reading speed in block 2. However, further research is needed to investigate if such a more global effect might exist.

The spillover region shows a somewhat different pattern of results than the verb. The two-way interaction between participant gender and block is found in both regions. The three-way interaction, however, was not found on the noun, nor was the main effect of gender marking. Instead, a two-way interaction between gender marking and block was found, showing that there was a tendency for both men and women to read the nouns following verbs with masculine marking faster, and the nouns following verbs with feminine marking slower, than the control nouns. Thus, there seems to be a processing disadvantage of verbs with feminine marking for both men and women which appears on the noun. For the female participants, this finding seems to be at odds with the effect found on the verb, which showed that verbs with feminine marking were read *faster* than the control verbs. How could the presence of these two effects be explained?

We should keep in mind that effects found on a spillover region in self-paced reading experiments can either reflect an early effect which spilled over onto the next word, or a late effect (Bicknell, Elman, Hare, McRae, Kutas, 2010). Thus, the difference between the effects found on the verb and on the noun could be explained in two different ways. Either, two different effects occurred more or less simultaneously during the processing of the verb, but only one of them showed up as an effect on the verb, while the other effect spilled over onto the noun. For example, it could be that on the one hand, the match between participant gender and gender marking facilitated processing, which showed up as the three-way interaction on the verb. At the same time, it is possible that both men and women are overall less exposed to feminine gender marking (when considering all kinds of contexts and referents) than masculine gender marking, which could result in a frequency effect (e.g., Kliegl, Grabner, Rolfs, Engbert, 2010; Mitchell & Green, 1978) that slows down the processing of verbs with feminine marking for both men and women. Such an effect could spill over onto the noun and show up as the two-way interaction between gender marking and block.

Alternatively, it is possible that gender marking has a different effect in the early and late stages of processing, and that the effect on the noun represents the late effect. For example, it could be that the process of pragmatic evaluation of the second-person gender marking is two-fold—first, the most immediate or salient context is evaluated, i.e. the addressee identity. Based on this information, both masculine marking in reference to women and feminine marking in reference to men is equally inappropriate, as shown by the similar effect on the verb for both men and women. Secondly, the broader context is taken into account, which may consider, for example, the other potential addressees, the author of the text, the genre of the text, together with more complex sociopragmatic conventions. During that process, both men and women may arrive at the interpretation that the use of masculine gender marking is felicitous given all this context, while the use of feminine gender marking is not—as reflected by the late processing disadvantage of feminine gender marking for both men and women. This type of effect might be similar to the late effects found by Jiang et al. (2013) in their study on honorifics. As mentioned before, the study found that both disrespectful and over-respectful uses elicited different late effects (late positivity and late negativity effects, respectively). The authors relate the late positivity effect to that of non-literal language processing (re-interpreting the over-respectful pronoun as constituting a joke or irony), and the late negativity effect to the mental revision or replacement of the disrespectful pronoun with the respectful one, after coming to the conclusion that its use was most likely a mistake.

It should be pointed out that both the ‘early and late effects’ and the ‘simultaneous effects’ interpretations of the results of the current study are highly speculative and require further investigation in future research. In fact, the speculativeness of the interpretations could be at least partially attributed to the limitations of the self-paced reading method, which cannot tell us as much about the nature and timing of an effect as ERPs. An ERP study could build on the current findings and provide more insights into the different effects that were found in the current study.

Another recommendation for future research would be to try to replicate the current study with gender marking as a between-subjects, rather than within-subjects, factor. It would be insightful to see whether the same pattern of results would hold in a between-subjects design. While this design would require a lot more participants to reach the same power, it would allow for a more straightforward analysis and interpretation of the results. It would eliminate the factor of block, meaning that the effect of gender marking could be considered without having to take into account practice effects between blocks. Additionally, the effect of feminine marking could be evaluated without the potentially confounding factor of *change* in gender marking between blocks. A between-subjects design could also clarify whether there is indeed a global effect of gender marking on reading time, or whether the two-way interaction between gender and block in the current study can rather be attributed to women being susceptible to larger practice effects. This could be done by, for example, comparing the reading time difference on fillers between the conditions to see whether it is positively affected by gender match and negatively by gender mismatch.

## Conclusion

Our results point to a processing disadvantage that women experience relative to men when addressed with verbs that have masculine gender marking. This is the first study, as far as we are aware, to provide evidence suggesting that masculine generics in the second person are biased in favour of male addressees during language processing and are not immediately perceived as gender-unspecific. The current findings thus add to the results of Kricheli-Katz and Regev (2021) who found that the use of Hebrew gender marking that mismatches addressee gender can negatively affect test performance. The results also suggest that the initial processing disadvantage that women experience when addressed with a masculine generic verb may be as strong as the effect of men being addressed through a verb with feminine gender marking. However, the results from the spillover region suggest that the effect of gender (mis)match is not the only one at play, as the reading times on the spillover region were faster for both men and women following verbs with masculine gender marking. We speculated that this could reflect a frequency effect or, alternatively, increased efforts in integrating feminine gender marking due to its markedness and lack of available generic reading. The current study is also the first one, to our knowledge, to provide evidence suggesting that mismatches between addressee identity and the linguistic form used can also negatively affect processing when the participant is the addressee, and that this disruption can show up in reading data. In this way, the current study adds to the ERP studies on the processing of mismatches between speaker or addressee identity, and the linguistic or semantic content of the text (e.g., Hanulíková & Carreiras, 2015; Jiang et al, 2013).

## Appendix

### Appendix A: List of stimuli (in the original Polish version and a translation) used in the self-paced reading experiment

Wyobraź sobie, że bierzesz udział w lekcji snowboardu. Gdy już przywiązałeś buty do deski, zamierzasz dołączyć do reszty grupy. Jednak w tym momencie podjeżdża do ciebie para narciarzy, którzy potrzebują pomocy w znalezieniu swojego hotelu.

‘Imagine that you are taking part in a snowboarding lesson. Once you tied your shoes to the board, you want to join the rest of the group. But then a pair of skiers who need help in finding their hotel come up to you.’

Wyobraź sobie, że wyjeżdżasz na wakacje w góry. Gdy już spakowałeś rzeczy do walizki, zamierzasz sprawdzić, o której godzinie odjeżdża pociąg. Jednak w tym momencie pojawiają się twoi rodzice z niespodziewaną wizytą.

‘Imagine that you are going on holiday in the mountains. Once you packed things in the suitcase, you want to check what time the train leaves. But then your parents show up with a surprise visit.’

Wyobraź sobie, że planujesz wyjście do kina wieczorem. Gdy już kupiłeś bilety przez internet, zamierzasz powiadomić o tym swoich przyjaciół. Jednak w tym momencie dostajesz wiadomość, że wszyscy zmienili zdanie i chcą obejrzeć inny film.

‘Imagine that you are planning to go to the cinema in the evening. Once you bought the tickets online, you want to let your friends know. But then you see a message that everyone has changed their minds and wants to see a different movie.’

Wyobraź sobie, że zdajesz egzamin na prawo jazdy. Gdy już zmieniłeś bieg na dwójkę, zamierzasz skręcić w boczną ulicę. Jednak w tym momencie słyszysz koło siebie chrapanie i orientujesz się, że egzaminator śpi.

‘Imagine that you are taking a driving exam. Once you changed the gear to the second one, you want to turn into a side road. But then you hear snoring next to you and you realise that the examiner is sleeping.’

Wyobraź sobie, że wyprowadzasz się do Norwegii na pół roku. Gdy już sprawdziłeś dojazd na lotnisko, zamierzasz pożegnać się z rodziną. Jednak w tym momencie babcia zaczyna pakować ci zapasy jedzenia do walizki.

‘Imagine that you are moving to Norway for half a year. Once you checked the transport to the airport, you want to say goodbye to your family. But then you see that your grandma is starting to pack food supplies into your suitcase.’

Wyobraź sobie, że biegniesz w półmaratonie po raz pierwszy w życiu. Gdy już przekroczyłeś metę po godzinach biegu, zamierzasz odebrać swój medal. Jednak w tym momencie podchodzi do ciebie dziennikarz z mikrofonem i zaczyna zadawać pytania.

‘Imagine that you are running a half-marathon for the first time in your life. Once you crossed the finish line after hours of running, you want to collect your medal. But then a journalist comes up to you with a microphone and starts asking you questions.’

Wyobraź sobie, że adoptujesz szczeniaka ze schroniska. Gdy już urządziłeś miejsce dla pieska w samochodzie, zamierzasz iść pożegnać się z pracownikami schroniska. Jednak w tym momencie pies wymyka się na przednie siedzenie i siusia prosto na twoją kurtkę.

‘Imagine that you are adopting a puppy from a shelter. Once you arranged a place for the dog in the car, you want to go say goodbye to the shelter employees. But then the dog sneaks out to the front seat and pees right on your jacket.’

Wyobraź sobie, że spędzasz niedzielę w parku krajobrazowym. Gdy już skręciłeś ścieżką w las, zamierzasz wyjąć aparat fotograficzny i zrobić kilka zdjęć. Jednak w tym momencie na twojej drodze staje lisica z pięcioma małymi lisami.

‘Imagine that you are spending a Sunday in a landscape park. Once you turned on a path leading into the forest, you want to take out your camera and take some pictures. But then a vixen with five little foxes appear on your path.’

Wyobraź sobie, że urządzasz imprezę sylwestrową w domu. Gdy już schowałeś szampana do lodówki, zamierzasz pojechać do sklepu po przekąski. Jednak w tym momencie widzisz, że pod twoimi drzwiami jest prawie pół metra śniegu.

‘Imagine that you are organising a New Year’s Eve party at home. Once you hid the champagne in the fridge, you want to go to a shop to buy snacks. But then you see that in front of your door there is almost half a meter of snow.’

Wyobraź sobie, że jesteś na kursie językowym we Włoszech. Gdy już zwiedziłeś miasto po zajęciach, zamierzasz poćwiczyć język z tubylcami w barze. Jednak w tym momencie uświadamiasz sobie, że większość osób wokół ciebie to brytyjscy turyści.

‘Imagine that you are at a language course in Italy. Once you went around the city after classes, you want to practice the language with the locals at a bar. But then you realise that most of the people around you are British tourists.’

Wyobraź sobie, że występujesz w sztuce teatralnej dla dzieci. Gdy już ubrałeś kostium w garderobie, zamierzasz szybko pójść do łazienki. Jednak w tym momencie uświadamiasz sobie, że w twoich spodniach jest wielka dziura.

‘Imagine that you are performing in a theatre play for children. Once you put on a costume in the dressing room, you want to quickly go to the bathroom. But then you realise that there is a huge hole in your pants.’

Wyobraź sobie, że spędzasz popołudnie w muzeum sztuki. Gdy już obejrzałeś kolekcję z dziełami dwudziestego wieku, zamierzasz przejść do specjalnej wystawy fotograficznej. Jednak w tym momencie słyszysz huk i widzisz, że jeden z obrazów zaczyna spadać.

‘Imagine that you are spending an afternoon in an art museum. Once you watched the collection with pieces from the 20th century, you want to go to the special photography exhibition. But then you hear a noise and you see that one of the paintings is starting to fall.’

Wyobraź sobie, że siedzisz w kawiarni niedaleko domu. Gdy już zjadłeś ciasto ze śliwkami, zamierzasz zamówić jeszcze jedną kawę. Jednak w tym momencie uświadamiasz sobie, że byłaby to już twoja piąta kawa tego dnia.’

‘Imagine that you are sitting in a cafe near home. Once you ate the plum cake, you want to order one more coffee. But then you realise that this would be your fifth coffee of the day.’

Wyobraź sobie, że szukasz nowych książek w księgarni. Gdy już przejrzałeś dział z literaturą obcą, zamierzasz wejść na drugie piętro. Jednak w tym momencie dostrzegasz najnowszą książkę twojego ulubionego autora.

‘Imagine that you are looking for new books in a bookshop. Once you looked through the section with foreign literature, you want to go up to the second floor. But then you notice the newest book by your favourite author.’

Wyobraź sobie, że spędzasz dzień na bałtyckiej plaży. Gdy już nałożyłeś krem do opalania, zamierzasz przejść się wzdłuż brzegu. Jednak w tym momencie podlatuje mewa i próbuje ukraść drożdżówkę z twojego plecaka.

‘Imagine that you are spending a day at a Baltic beach. Once you put on sunscreen, you want to go for a walk along the shore. But then a seagull flies up and tries to steal a pastry from your backpack.’

Wyobraź sobie, że zwiedzasz wielkie oceanarium. Gdy już zobaczyłeś zwierzęta z Afryki Wschodniej, zamierzasz skierować się do wyjścia. Jednak w tym momencie przebudza się mały hipopotam i zaczyna się bawić w wodzie, więc zostajesz na niego popatrzeć.

‘Imagine that you are visiting a big oceanarium. Once you saw the animals from East Africa, you want to head towards the exit. But then a little hippo wakes up and starts playing in the water, so you stay to watch it.’

Wyobraź sobie, że jesteś w samolocie lecącym do Stanów Zjednoczonych. Gdy już włożyłeś torbę pod siedzenie, zamierzasz włączyć tryb samolotowy w komórce. Jednak w tym momencie słyszysz ogłoszenie, że jest problem z silnikiem i nie wiadomo, kiedy samolot będzie mógł wystartować.

‘Imagine that you are on a plane to the United States. Once you put your suitcase under the seat, you want to turn on airplane mode on your phone. But then you hear an announcement that there is a problem with the engine and it is not known when the plane will be able to take off.’

Wyobraź sobie, że jesteś w restauracji greckiej z całą rodziną. Gdy już zamówiłeś zupę z soczewicy, zamierzasz przejrzeć kartę win. Jednak w tym momencie podchodzi kelner i daje wam butelkę wina na koszt restauracji.

‘Imagine that you are at a Greek restaurant with the whole family. After you ordered a lentil soup, you want to look through the wine card. But then the waiter comes by and gives you a bottle of wine on the house.’

Wyobraź sobie, że pracujesz w popularnym barze w sobotni wieczór. Gdy już zaniosłeś drinki dla pary siedzącej na zewnątrz, zamierzasz obsłużyć klientów czekających przy barze. Jednak w tym momencie dwie osoby z kolejki zaczynają się bić.

‘Imagine that you are working at a popular bar on a Saturday evening. Once you brought drinks for a couple sitting outside, you want to serve clients waiting at the bar. But then two people in the line begin to fight.’

Wyobraź sobie, że jedziesz autobusem do centrum miasta. Gdy już skasowałeś bilet przy wejściu, zamierzasz znaleźć miejsce do siedzenia. Jednak w tym momencie autobus gwałtownie hamuje i spadasz prosto na kolana starszej pani.

‘Imagine that you are taking the bus to the city centre. Once you punched your ticket at the entrance, you want to find a place to sit. But then the bus breaks suddenly and you fall straight on an old lady’s lap.’

Wyobraź sobie, że zaczynasz staż w międzynarodowej firmie. Gdy już podpisałeś umowę z pracodawcą, zamierzasz iść poznać członków twojego zespołu. Jednak w tym momencie uruchamia się alarm pożarowy i wszyscy kierują się do wyjścia.

‘Imagine that you are starting an internship at an international company. Once you signed the contract with your employer, you want to go meet the members of your team. But then a fire alarm starts and everyone heads towards the exit.’

Wyobraź sobie, że jesteś na swojej pierwszej lekcji gotowania. Gdy już upiekłeś mięso w piekarniku, zamierzasz przyrządzić sos. Jednak w tym momencie słyszysz krzyk i widzisz, że osoba koło ciebie zraniła się nożem.

‘Imagine that you are in your first cooking class. Once you baked the meat in the oven, you want to prepare the sauce. But then you hear a scream and see that the person next to you has hurt themselves with a knife.’

Wyobraź sobie, że wspinasz się na szczyt górski na wakacjach w Chorwacji. Gdy już wyciągnąłeś mapę z plecaka, zamierzasz udać się w stronę szlaku. Jednak w tym momencie uświadamiasz sobie, że nie masz ze sobą nic do picia.

‘Imagine that you are climbing a mountain on holiday in Croatia. Once you took out the map from your backpack, you want to head towards the trail. But then you realise that you don’t have anything to drink with you.’

Wyobraź sobie, że jesteś na jarmarku bożonarodzeniowym. Gdy już wypiłeś grzańca do dna, zamierzasz pooglądać ozdoby na choinkę. Jednak w tym momencie widzisz parę przyjaciół, więc idziesz przywitać się z nimi.

‘Imagine that you are at a Christmas market. Once you drank all of the mulled wine you want to look at the Christmas tree decorations. But then you see a pair of your friends, so you go to say hi to them.’

Wyobraź sobie, że jesteś na łyżwach ze znajomymi. Gdy już okrążyłeś lodowisko bez trudu, zamierzasz spróbować kilku sztuczek. Jednak w tym momencie wszyscy są proszeni o opuszczenie lodowiska, bo ma się na nim rozpocząć mecz hokeja.

‘Imagine that you are ice skating with your friends. Once you circled the ice rink without trouble, you want to try out some tricks. But then everyone is asked to leave the ice rink, because a hockey match is about to start on it.’

Wyobraź sobie, źe jesteś na spływie kajakowym po Odrze. Gdy już odebrałeś wiosła z wypożyczalni, zamierzasz udać się w stronę rzeki. Jednak w tym momencie zaczyna padać grad, więc czekasz w środku aż przestanie.

‘Imagine that you are on a kayaking trip down the Odre. Once you picked up the paddles from the rental place, you want to head towards the river. But then it starts hailing so you wait inside until it stops.’

Wyobraź sobie, że jesz śniadanie na tarasie w ogródku. Gdy już nalałeś kawy do filiżanki, zamierzasz posmarować chleb masłem. Jednak w tym momencie pies sąsiada wbiega do ogródka i zaczyna wpatrywać się w twój talerz.

‘Imagine that you are eating breakfast on a garden terrace. Once you poured coffee into the cup, you want to butter your bread. But then your neighbour’s dog runs into the garden and starts staring at your plate.’

Wyobraź sobie, że jedziesz pociągiem do Warszawy. Gdy już pokazałeś bilet do kontroli, zamierzasz założyć słuchawki na uszy i trochę pospać. Jednak w tym momencie dziecko siedzące naprzeciwko zaczyna cię kopać.

‘Imagine that you are going by train to Warsaw. Once you showed your ticket to be checked, you want to put on headphones and sleep for a bit. But then a child sitting across from you starts kicking you.’

Wyobraź sobie, że jesteś na rejsie po kanałach Amsterdamu. Gdy już wysłałeś zdjęcie do rodziców, zamierzasz przesiąść się na miejsce w cieniu. Jednak w tym momencie łódka zaczyna się chwiać i wpadasz do kanału.

‘Imagine that you are on a boat tour in the canals of Amsterdam. Once you sent a picture to your parents, you want to move to a seat in the shade. But then the boat starts rocking and you fall into the canal.’

Wyobraź sobie, że jesteś w kinie plenerowym. Gdy już przyniosłeś popcorn dla grupy znajomych, zamierzasz rozsiąść się na leżaku. Jednak w tym momencie zaczyna mocno wiać i wichura porywa twój leżak.

‘Imagine that you are at an open air cinema. Once you brought popcorn for a group of friends, you want to settle down on the chair. But then a heavy wind starts blowing and blows your chair away.’

Wyobraź sobie, że jesteś na poczcie, aby wysłać kartkę z życzeniami. Gdy już nakleiłeś znaczek na kopertę, zamierzasz napisać adres odbiorcy. Jednak w tym momencie osoba, do której wysyłasz kartkę pojawia się na poczcie.

‘Imagine that you are at the post office to send a greetings card. Once you glued a stamp onto the envelope, you want to write the address on it. But then the person who you are sending the card to appears at the post office.’

Wyobraź sobie, że montujesz szafkę z Ikei w sypialni. Gdy już rozłożyłeś części na dywanie, zamierzasz powkładać kołki w otwory. Jednak w tym momencie słyszysz dzwonek do drzwi, i przypominasz sobie, że masz gości na dzisiejszy wieczór.

‘Imagine that you are assembling a cabinet from Ikea in the bedroom. Once you put down the parts on the carpet, you want to start putting the pegs in the holes. But then you hear the doorbell and you remember that you are having guests over tonight.’

Wyobraź sobie, że nagrywasz krótki film podczas rodzinnej wycieczki do Krakowa. Gdy już włączyłeś kamerę przy zamku na Wawelu, zamierzasz sfilmować go ze wszystkich stron. Jednak w tym momencie podchodzi twoja mama i zaczyna wygłupiać się przed kamerą.

‘Imagine that you are making a short video during a family trip to Kraków. Once you turned on the camera near the Wawel castle, you want to film it from every side. But then your mum comes up and starts acting silly in front of the camera.’

Wyobraź sobie, że siedzisz przy ognisku w lesie na biwaku. Gdy już usmażyłeś kiełbasę na ogniu, zamierzasz nałożyć sałatkę na talerz. Jednak w tym momencie z lasu wyłania się nieznana osoba i kieruje się w twoją stronę.

‘Imagine that you are sitting by a fire in a forest on a camping trip. Once you fried a sausage on the fire, you want to put some salad on the plate. But then a strange person emerges from the forest and comes in your direction.’

Wyobraź sobie, że spędzasz dzień w parku rozrywki. Gdy już zaliczyłeś przejażdżkę po wodospadach, zamierzasz stanąć w kolejce do diabelskiego młynu. Jednak w tym momencie znajdujesz na ziemi czyjś portfel, więc postanawiasz zanieść go do punktu znalezionych rzeczy.

‘Imagine that you are in an amusement park for the whole day. Once you did a ride through waterfalls, you want to stand in line for the ferris wheel. But then you find someone’s wallet on the ground so you decide to bring it to the lost and found point.’

Wyobraź sobie, że planujesz obejrzeć ważny mecz siatkówki w domu. Gdy już przełączyłeś kanał na telewizorze, zamierzasz usadowić się na kanapie. Jednak w tym momencie w całym domu następuje awaria prądu.

‘Imagine that you are planning to watch an important volleyball match at home. Once you swtiched the channel on the tv, you want to settle down on the couch. But then the power suddenly goes off in the whole house.’

Wyobraź sobie, że próbujesz nauczyć się grać na gitarze. Gdy już poćwiczyłeś akordy przez godzinę, zamierzasz znaleźć filmik instruktażowy do łatwej piosenki. Jednak w tym momencie zaczynasz się zastanawiać, czy twoja gitara jest na pewno dobrze nastrojona.

‘Imagine that you are trying to learn to play guitar. Once you practiced the chords for an hour, you want to find a video tutorial for an easy song. But then you start to wonder whether the guitar is really well tuned.’

Wyobraź sobie, że zapisujesz się na zajęcia w siłowni w twojej okolicy. Gdy już wypełniłeś formularz w recepcji, zamierzasz iść się przebrać w szatni. Jednak w tym momencie podchodzi do ciebie pracownik i oferuje cię oprowadzenie cię po siłowni.

‘Imagine that you are signing up for classes at the local gym. Once you filled out the form at the reception, you want to go change in the changing room. But then an employee comes up to you and offers to show you around the gym.’

Wyobraź sobie, że wynajmujesz pokój gościnny w domku nad jeziorem. Gdy już zapłaciłeś kaucję w dzień przyjazdu, zamierzasz przejść się po okolicy. Jednak w tym momencie gospodarze domu częstują cię kawą i ciastem.

‘Imagine that you are renting a guest room in a house by a lake. Once you paid the deposit on the day of the arrival, you want to take a walk around the area. But then the hosts offer you coffee and cake.’

Wyobraź sobie, że przygotowujesz drinki dla gości na imprezie. Gdy już wymieszałeś składniki w shakerze, zamierzasz znaleźć odpowiednie szklanki. Jednak w tym momencie dociera do ciebie, że zamiast cukru w miksturze przypadkowo znalazła się sól.

‘Imagine that you are making cocktails for guests at a party. Once you mixed the ingredients in a shaker, you want to find the right glasses. But then you realise that instead of sugar, salt accidentally ended up in the mixture.’

Wyobraź sobie, że obserwujesz niebo w bezchmurną noc w górach. Gdy już wypatrzyłeś Wenus wśród gwiazd, zamierzasz sięgnąć po lornetkę i znaleźć kilka gwiazdozbiorów. Jednak w tym momencie ktoś podchodzi do ciebie i pyta, czy może pożyczyć twoją lornetkę.

‘Imagine that you are looking at the sky on a cloudless night in the mountains. Once you spotted Venus among the starts, you want to take the binoculars and find a few constellations. But then someone up to you and asks if they can borrow your binoculars.’

Wyobraź sobie, że jesteś z rodziną na grzybach w pobliskim lesie. Gdy już nabrałeś kurek do koszyka, zamierzasz udać się w stronę samochodu. Jednak w tym momencie słyszysz radosny krzyk twojej cioci, która znalazła miejsce pełne prawdziwków.

‘Imagine that you are picking mushrooms with your family in a nearby forest. Once you collected chanterelles into the basket, you want to head towards the car. But then you hear a joyful cry from your aunt who found a spot full of porcinos.’

Wyobraź sobie, że montujesz karmnik dla ptaków na zimę. Gdy już powiesiłeś domek na balkonie, zamierzasz wsypać do niego pokarm. Jednak w tym momencie podlatują dwie sikorki i próbują dostać się do torby z jedzeniem.

‘Imagine that you are installing a bird feeder for the winter. Once you hung the house on the balcony, you want to put food into it. But then two tits fly by and try to get inside of the bag of food.’

Wyobraź sobie, że jesteś na basenie w hotelu. Gdy już zamoczyłeś stopy w wodzie, zamierzasz wskoczyć i zanurkować. Jednak w tym momencie w rogu basenu widzisz brudną pieluchę.

‘Imagine that you are at a hotel pool. Once you wet your feet in the water, you want to jump inside and dive. But then in the corner of the pool you see a dirty diaper.’

Wyobraź sobie, że jesteś na nartach z grupą przyjaciół. Gdy już wjechałeś wyciągiem na szczyt góry, zamierzasz zrobić zdjęcie krajobrazu. Jednak w tym momencie wjeżdża w ciebie osoba wysiadająca z wyciągu i razem lecicie na dół.

‘Imagine that you are on a skiing trip with a group of friends. Once you rode the ski lift to the top of the mountain, you want to take a picture of the view. But then a person exiting the lift skis into you and you both start going down the hill.’

Wyobraź sobie, że uczestniczysz w pierwszym wykładzie na nowym kierunku studiów. Gdy już otworzyłeś notatnik na laptopie, zamierzasz zapisać kilka ważnych informacji. Jednak w tym momencie z twojej przeglądarki zaczyna grać na cały głos reklama z otwartej strony internetowej.

‘Imagine that you are attending the first lecture of a new university programme. Once you opened a notebook on your laptop, you want to write down some important information. But then an ad starts playing at full volume from an open website on your browser.’

Wyobraź sobie, że bierzesz udział w kursie spadochronowym. Gdy już przypiąłeś spadochron w samolocie, zamierzasz wykonać swój pierwszy skok. Jednak w tym momencie twój instruktor oświadcza, że źle się czuje i nie wie, czy będzie w stanie z tobą skoczyć. 94.

‘Imagine that you are taking part in a sky diving course. Once you attached the parachute on the plane, you want to make you first dive. But then your instructor announces that he is not feeling well and he does not know whether he will be able to jump with you.’

Wyobraź sobie, że przemeblowujesz swój pokój. Gdy już przesunąłeś biurko pod okno, zamierzasz postawić na nim lampkę. Jednak w tym momencie orientujesz się, że nie ma w pobliżu żadnego gniazdka.

‘Imagine that you are rearranging your room. After moving the desk next to the window, you want to put a lamp on it. But then you realise that there is no power socket near it.’

### Appendix B: The mean reading times per condition in the self-paced reading experiment

See Tables

**Table 4:** Mean reading times (in ms) of the verb

Gender marking	Block 1 (gender marking = masculine)				Block 2 (gender marking = feminine)			
	Participant gender				Participant gender			
	Female		Male		Female		Male	
	M	SD	M	SD	M	SD	M	SD
Yes	650	400	573	305	491	285	545	346
No	661	393	606	319	515	302	522	276

^4^,

**Table 5:** Mean reading times (in ms) of the noun following the verb

Gender marking	Block 1 (gender marking = masculine)				Block 2 (gender marking = feminine)			
	Participant gender				Participant gender			
	Female		Male		Female		Male	
	M	SD	M	SD	M	SD	M	SD
Yes	661	408	603	342	527	318	556	317
No	666	425	626	366	488	261	528	262

^5^.

### Appendix C: The output of the linear models fitted to the data from the self-paced reading experiment

See Tables

**Table 6:** Output of the linear mixed model conducted on the main region of interest (verb)

	Estimate	Standard error	t-value	*P*-value
Intercept	6.2143	0.0421	147.3164	0.0000
gendermarkingnovyes	− 0.0156	0.0050	− 3.1456	0.0017**
blockonevtwo	− 0.0889	0.0120	− 7.4335	0.0000***
ppgenderfvm	0.0041	0.0421	0.0972	0.9228
gendermarkingnovyes:blockonevtwo	0.0056	0.0050	1.1256	0.2604
gendermarkingnovyes:ppgenderfvm	− 0.0043	0.0050	− 0.8655	0.3868
blockonevtwo:ppgenderfvm	− 0.0344	0.0120	− 2.8746	0.0052**
gendermarkingnovyes:blockonevtwo:ppgenderfvm	− 0.0114	0.0050	− 2.2897	0.0221*

^*^*P* ≤ 0.05, ***P* ≤ 0.01, ****P* ≤ 0.0001

*ppgender* participant gender, *f* female, *m* male

6,

**Table 7:** Output of the linear mixed model conducted on the spillover region (noun)

	Estimate	Standard error	t-value	*P*-value
Intercept	6.2347	0.0415	150.2871	0.0000
gendermarkingnovyes	0.0070	0.0051	1.3641	0.1726
blockonevtwo	− 0.0880	0.0106	− 8.2922	0.0000***
ppgenderfvm	− 0.0072	0.0412	− 0.1752	0.8614
gendermarkingnovyes:blockonevtwo	0.0161	0.0051	3.1435	0.0017**
gendermarkingnovyes:ppgenderfvm	0.0070	0.0061	1.1551	0.2536
blockonevtwo:ppgenderfvm	− 0.0308	0.0106	− 2.9034	0.0048**
gendermarkingnovyes:blockonevtwo:ppgenderfvm	− 0.0010	0.0051	− 0.2006	0.8410

^*^*P* ≤ 0.05, ***P* ≤ 0.01, ****P* ≤ 0.0001

*ppgender* participant gender, *f* female, *m* male

7.

## Footnotes

1 This rule is sometimes challenged by non-binary speakers, but is very much considered non-standard. Additionally, perhaps due to the association of neuter gender with inanimate objects, animals and children, non-binary speakers often prefer other ways of expressing their gender linguistically rather than adopting the neuter gender marking (Dziura, 2018).

2 Abbreviations used in the glosses: 2 = second person; acc = accusative case; f = feminine gender; gen = genitive case; m = masculine gender; sg = singular.
